# Supplementary material for: Variations in Postmenopausal Body Composition: A Cross-Sectional Comparison between Physical Activity Practitioners and Sedentary Individuals
Source: J Funct Morphol Kinesiol. 2023 Dec 30;9(1):12. doi: 10.3390/jfmk9010012 (PMC10801510; doi:10.3390/jfmk9010012)
Supplement: Supplementary file 1 [file jfmk-09-00012-s001.zip › jfmk-2715975-supplementary.pdf]

Table S1. Anthropometric variables of all women

VARIABLE	PHYSICAL ACTIVITY		P VALUE	PR (CI 95%)
	ACTIVE N (%)	SEDENTARY N (%)		
**BMI**				
Normal	116 (31.44)	99 (31.94)		0.95 (0.79;1.14)
Overweight	131 (35.50)	102 (32.90)	0.755	1.02 (0.85;1.22)
Obesity	122 (33.06)	109 (35.16)		
**FMI**				
Normal	78 (21.43)	59 (19.47)		
Overweight	140 (38.46)	121 (39.93)	0.815	1.04 (0.90;1.20)
Obesity	146 (40.11)	123 (940.59)		1.03 (0.90;1.19)
**WC**				
Risk-free	123 (33.06)	96 (30.67)		
High risk	105 (28.23)	83 (26.52)	0.552	1.00 (0.81;1.24)
Very high risk	144 (38.71)	134 (42.81)		1.08 (0.92;1.26)
**WHtR**				
Low risk	113 (30.38)	96 (30.67)		
High risk	259 (69.62)	217 (69.33)	0.934	0.99 (0.90;1.10)
**WHR**				
Baixo risco	94 (25.27)	79 (25.24)		
Alto risco	278 (74.73)	234 (74.76)	0.993	1.00 (0.91;1.09)
**Bone mass**				
Normal	155 (44.67)	135 (45.92)	0.331	
Osteopenia	111 (31.99)	104 (35.37)		1.04 (0.85;1.27)
Osteoporosis	81 (23.34)	55 (18.71)		0.84 (0.63;1.12)
**Appendicular Lean Mass**				
	53 (14.29)	51 (16.29)	0.466	1.14 (0.80;1.62)
Low Discharge	318 (85.71)	262 (83.71)		

Table S2. Body indices derived from anthropometric and DEXA measurements of women who have not gone through menopause.

VARIABLE	PHYSICAL ACTIVITY		P VALUE	PR (CI 95%)
	ACTIVE N (%)	SEDENTARY N (%)		
**BMI**	82 (36.61)	76 (42.94)		0.73 (0.55;0.97)
NORMAL	80 (35.71)	43 (24.29)	0.048	1.00 (0.76;1.31)
Overweight	62 (27.68)	58 (32.77)		
Obesity				

**FMI**				
Normal	59 (27.06)	45 (26.01)		0.99 (0.79;1.24)
Overweight	77 (35.32)	58 (33.53)	0.847	1.04 (0.85;1.28)
Obesity	82 (37.61)	70 (40.46)		
**WC**				
Risk-free	92 (40.89)	81 (45.00)		0.85 (0.63;1.16)
High risk	64 (28.44)	44 (24.44)	0.608	0.94 (0.71;1.23)
Very high risk	69 (30.67)	55 (30.56)		
**WHtR**				
Low risk	91 (40.44)	86 (47.78)	0.139	0.87 (0.73;1.04)
High risk	134 (59.56)	94 (52.22)		
**WHR**				
Baixo risco	83 (36.89)	72 (40)	0.522	0.95 (0.81;1.11)
Alto risco	142 (63.11)	108 (60)		
**Bone mass**				
Normal	122 (60.10)	89 (54.60)		
Osteopenia	48 (23.65)	65 (39.88)	0.000	1.49 (1.10;2.02)
Osteoporosis	33 (16.26)	9 (5.52)		0.43 (0.21;0.86)
**Appendicular Lean Mass**				
Low	31 (13.84)	35 (19.44)	0.130	1.40 (0.90;2.18)
Discharge	193 (86.16)	145 (80.56)		

Table S3. Anthropometric variables of postmenopausal women

**VARIABLE**	**PHYSICAL ACTIVITY**		**P VALUE**	**PR (CI 95%)**
	**ACTIVE** N (%)	**SEDENTARY** N (%)		
**BMI**	34 (23.45)	23 (17.29)		
Normal	51 (35.17)	59 (44.36)	0.232	1.19 (0.96;1.49)
Overweight	60 (41.38)	51 (38.35)		1.07 (0.87;1.33)
Obesity				
**FMI**				
Normal	19 (13.01)	14 (10.77)		
Overweight	63 (43.15)	63 (48.46)	0.648	1.06 (0.90;1.24)
Obesity	64 (43.84)	53 (40.77)		1.02 (0.86;1.21)
**WC**				
Risk-free	31 (21.09)	15 (11.28)		
High risk	41 (27.89)	39 (29.32)	0.081	1.26 (0.97;1.64)
Very high risk	75 (51.02)	79 (59.40)		1.18 (1.02;1.38)
**WHtR**	22 (14.97)	10 (7.52)		
Low risk	125 (85.03)	123 (92.48)	0.050	1.08 (1.00;1.18)
High risk				
**WHR**				
Baixo risco	11 (7.48)	7 (5.26)	0.449	1.02 (0.96;1.08)

Alto risco	136 (92.52)	126 (94.74)		
**Bone mass**				
Normal	33 (22.92)	46 (35.11)		
Osteopenia	63 (43.75)	39 (29.77)	0.027	0.69 (0.53;0.91)
Osteoporosis	48 (33.33)	46 (35.11)		0.84 (0.64;1.10)
**Appendicular Lean Mass**				
	22 (14.97)	16 (12.03)		
Low Discharge	125 (85.03)	117 (87.97)	0.474	0.80 (0.44;1.46)

Table S4. Anthropometric variables of physiological, premature, and early postmenopausal women.

Variable	PHYSIOLOGICAL PHYSICAL ACTIVITY		P VALUE	PREMATURE PHYSICAL ACTIVITY		P VALUE	PRECOCIOUS PHYSICAL ACTIVITY		P VALUE
	ACTIVE N (%)	SEDENTARY N (%)		ACTIVE N (%)	SEDENTARY N (%)		ACTIVE N (%)	SEDENTARY N (%)	
**BMI**		11(15.07)							
Normal	20(25.00)	34 (46.58)	0.177	4(16.67)	2(9.09)	0.336	10(24.39)	10(26.32)	
Overweight	27(33.75)	28 (38.36)		12(50.00)	8(36.36)		12(29.27)	17(44.74)	0.248
Obesity	33(41.25)			8(33.33)	12(54.55)		19(46.34)	11(28.95)	
**FMI**		6(8.33)							
Normal	9(11.25)	35(48.61)	0.770	4(16.67)	1(5.00)	0.423	5(12.20)	7(18.42)	
Overweight	35(43.75)	31(43.06)		10(41.67)	8(40.00)		18(43.90)	20(52.63)	0.402
Obesity	36(45.00)			10(41.67)	11(55.00)		18(43.90)	11(28.95)	
**WC**		10(13.70)							
Risk-free	16(20.00)	42(57.53)	0.407	6(25.00)	0(0.00)	0.032	7(17.07)	5(13.16)	
High risk	38(47.50)	21(28.77)		12(50.00)	17(77.27)		25(60.98)	20(52.63)	0.519
Very high risk	26(32.50)			6(25.00)	5(22.73)		9(21.95)	13(34.21)	
**WHtR**		7(9.59)							
Low risk	11(13.75)	66(90.41)	0.462	3(12.50)	0(00.00)	0.086	6(14.63)	3(7.89)	
High risk	69(86.25)			21(87.50)	22(100.00)		35(85.37)	35(92.11)	0.346
**WHR**		5(6.85)							
Low	5 (6.25)	68(93.15)	1.000	1(4.17)	0(0.00)	0.333	3(7.32)	2(5.26)	
High risk	75(93.75)			23(95.83)	22(100.00)		38(92.68)	36(94.74)	0.708

**Bone mass**									
Normal	15(18.99)	25(34.72)	0.084	9(39.13)	13(59.09)	0.345	9(22.50)	8(21.62)	0.167
Osteopenia	37(46.84)	25(34.72)		8(34.78)	4(18.18)		18(45.00)	10(27.03)	
Osteoporosis	27(34.18)	22(30.56)		6(26.09)	5(22.73)		13(32.50)	19(51.35)	
**Appendicular lean mass**									
Low	11(13.75)	8(10.96)	0.633	3(12.50)	1(4.55)	0.339	6(14.63)	7(18.42)	0.765
Discharge	69(86.25)	65(89.04)		21(87.50)	21(95.45)		35(85.37)	31(81.58)	

Table S5. Prevalence ratio adjusted for age, race and skin color, of the overall sample.

Variable	PR (CI 95%)*	PR (CI 95%)**	PR (CI 95%)***
**BMI overweight**	0.99 (0.89;1.09)	0.97 (0.87;1.07)	0.99 (0.89;1.09)
**BMI obesity**	1.11 (0.89;1.32)	1.02 (0.82;1.22)	1.11(0.89;1.32)
**FMI overweight**	1.16 (0.91;1.41)	1.07 (0.84;1.31)	1.16 (0.91;1.41)
**FMI obesity**	1.05 (0.92;1.17)	1.02 (0.90;1.14)	1.04 (0.92;1.16)
**WC High risk**	1.09 (0.86;1.33)	1.00 (0.78;1.22)	1.09 (0.85;1.32)
**WC Very high risk**	1.12 (0.85;1.32)	1.04 (1.01;1.24)	1.12 (0.94;1.15)
**WHtR**	1.11 (1.00;1.24)	0.99 (0.91;1.31)	1.11 (0.82;1.16)
**WHR**	1.12 (0.91;1.31)	1.00 (0.91;1.32)	1.12 (0.81;1.19)
**Bone mass osteopenia**	1.12 (0.91;1.33)	1.04 (0.92;1.32)	1.12 (0.85;1.22)
**Bone mass osteoporosis**	0.95 (0.92;1.32)	0.93 (0.82;1.07)	0.94 (0.82;1.04)
**Lean Mass Index**	1.08 (0.82;1.07)	1.08 (0.85;1.31)	1.07 (0.84;1.30)

Table S6. Prevalence ratio adjusted for age, race and skin color, of women who did not go through menopause.

Variável	PR (CI 95%)*	PR (CI 95%)**	PR (CI 95%)***
**BMI overweight**	0.88 (0.76;1.01)	0.86 (0.74;0.98)	0.88 (0.76;1.01)
**BMI obesity**	1.11 (0.85;1.37)	1.00 (0.76;1.24)	1.11 (0.85;1.37)
**FMI overweight**	1.13 (0.82;1.44)	0.99 (0.71;1.29)	1.13 (0.82;1.44)
**FMI obesity**	1.05 (0.90;1.20)	1.02 (0.88;1.17)	1.04 (0.90;1.19)
**WC High risk**	0.96 (0.70;1.23)	0.86 (0.63;1.12)	0.95 (0.70;1.23)
**WC Very high risk**	1.04 (0.91;1.18)	0.97 (0.85;1.09)	1.04 (0.91;1.18)
**WHtR**	0.97 (0.75;1.20)	0,84 (0.65;1.04)	0.97 (0.75;1.19)
**WHR**	1.05 (0.82;1.28)	0.92 (0.72;1.14)	1.05 (0.82;1.28)
**Bone mass osteopenia**	1.52 (1.23;1.78)	1.36 (1.09;1.62)	1.52 (1.23;1.78)
**Bone mass osteoporosis**	0.82 (0.60;1.06)	0.73 (0.54;0.94)	0.82 (0.60;1.06)
**Lean Mass Index**	1.23 (0.92;1.53)	1.23 (0.93;1.53)	1.23 (0.92;1.53)

* Adjusted for age; ** Adjusted by Race/Color; ***Adjusted for age and race/color

Table S7. Prevalence ratio adjusted for age and race, skin color, and women who have already gone through menopause.

Variable	PR (CI 95%)*	PR (CI 95%)**	PR (CI 95%)***
**BMI overweight**	0.88 (0.76;1.01)	0.86 (0.74;0.98)	0.88 (0.76;1.01)
**BMI obesity**	1.13 (0.75;1.53)	1.18 (0.79;1.58)	1.18 (0.79;1.58)
**FMI overweight**	1.17 (0.74;1.61)	1.18 (0.75;1.62)	1.18 (0.75;1.62)
**FMI obesity**	1.03 (0.81;1.26)	1.04 (0.82;1.27)	1.04 (0.82;1.27)
**WC High risk**	1.49 (0.94;2.05)	1.51 (0.95;2.07)	1.52 (0.96;2.08)
**WC Very high risk**	1.29 (1.03;1.55)	1.28 (1.03;1.55)	1.29 (1.04;1.56)
**WHtR**	1.60 (1.00;2.20)	1.64 (1.03;2.23)	1.65 (1.04;2.25)
**WHR**	1.26 (0.68;1.85)	1.25 (0.67;1.84)	1.28 (0.69;1.87)
**Bone mass osteopenia**	0.67 (0.44;0.93)	0.65 (0.43;0.90)	0.67 (0.43;0.93)
**Bone mass osteoporosis**	0.92 (0.79;1.05)	0.91 (0.78;1.04)	0.92 (0.79;1.05)
**Lean Mass Index**	0.87 (0.55;1.23)	0.84 (0.52;1.20)	0.85 (0.53;1.20)

* Adjusted for age; ** Adjusted by Race/Color; ***Adjusted for age and race/color
